# Baseline Susceptibility and Diagnostic Concentration of AcMNPV in *Rachiplusia nu* (Lepidoptera: Noctuidae) in Brazil and Cross-resistance to Cry1Ac

**DOI:** 10.1007/s13744-026-01374-x

**Published:** 2026-03-13

**Authors:** Arthur Dallanora, Daniela Neves Godoy, Venicius Ernesto Pretto, Otavio Liberalesso, Luiz Francisco Warpechowski, Oderlei Bernardi

**Affiliations:** Dept of Plant Protection, Federal Univ of Santa Maria, Santa Maria, RS Brazil

**Keywords:** Sunflower looper, Biological control, Baculovirus, Resistance management

## Abstract

The sunflower looper, *Rachiplusia nu* (Guenée, 1852) (Lepidoptera: Noctuidae), has evolved practical resistance to the Cry1Ac toxin from *Bacillus thuringiensis* Berliner (*Bt*) expressed in soybean in Brazil and neighboring countries. In view of this, the use of chemical or biological insecticides is required for their control. In 2023, a bioinsecticide based on *Autographa californica* multiple nucleopolyhedrovirus (AcMNPV: Baculoviridae: Alphabaculovirus) was registered for the control of *R. nu* in Brazil. To support the use of this new insecticide, we conducted diet-overlay bioassays to characterize the baseline susceptibility of Brazilian populations of *R. nu* to AcMNPV and establish a diagnostic concentration for resistance monitoring. Additionally, we evaluated cross-resistance between AcMNPV and Cry1Ac by testing AcMNPV in both Cry1Ac-susceptible and -resistant populations of *R. nu*. The tested field populations of *R. nu* were susceptible to AcMNPV. The LC_50_ of AcMNPV ranged from 1.9 × 10^7^ to 7.9 × 10^7^ occlusion bodies (OBs)/mL, indicating a low (< 4.2-fold) interpopulation variation in susceptibility. The diagnostic concentration of AcMNPV established, based on the calculated LC_99_ (1.6 × 10^9^ OBs/mL), caused > 97% mortality in field populations of *R. nu*. No cross-resistance was detected between AcMNPV and Cry1Ac *Bt* toxin in this species. These findings indicate that the AcMNPV-based bioinsecticide may be a valuable tool in integrated management programs to control *R. nu* and that the diagnostic concentration determined here is suitable for resistance monitoring.

## Introduction

The sunflower looper, *Rachiplusia nu* (Guenée) (Lepidoptera: Noctuidae), was historically categorized as a secondary soybean (*Glycine max* [L.] Merrill) pest in Brazil (Guedes et al. 2015; Horikoshi et al. 2021a). However, during the 2020/21 soybean season, *R. nu* evolved practical resistance to the soybean MON 87701 × MON 89788, which expresses Cry1Ac toxin from *Bacillus thuringiensis* Berliner (*Bt*); this resistance is currently reported in all soybean-producing regions of Brazil (Horikoshi et al. 2021b; Reis et al. 2024; Godói et al. 2025). In the 2021/22, *R. nu* also evolved resistance to Cry1Ac *Bt* soybean in Argentina, where it is a primary soybean pest (Hill et al. 2023).

The use of chemical insecticides has been the main tactic for controlling *R. nu* in Cry1Ac soybean in Brazil and neighboring countries. Recent reports indicated that field populations of *R. nu* from Brazil have a high susceptibility to chemical insecticides (Braga et al. 2024; Godoy et al. 2024). However, some Brazilian populations presented moderate levels of resistance to flubendiamide and bifenthrin, as did Argentinian populations to pyrethroids (Rimoldi et al. 2012; Russo et al. 2012; Godoy et al. 2024). In this situation, the frequent use of chemical insecticides against *R. nu* may favor the evolution of resistance if appropriate management practices are not adopted.

In 2023, an *Autographa californica* multiple nucleopolyhedrovirus (AcMNPV)–based bioinsecticide, an insecticide with a new mode of action (Group 31, Insecticide Resistance Action Committee [IRAC]), was registered in Brazil for controlling *R. nu* and *Helicoverpa armigera* (Hübner) (Lepidoptera: Noctuidae). Baculovirus-based insecticides stand out as an alternative to chemical insecticides, being a valuable control option for soybean pests because of their effectiveness, specificity, selectivity to non-target organisms, and safety to humans and the environment (Moscardi 1999).

Insecticides based on baculoviruses are also useful for insect resistance management programs. Previous studies have reported no cross-resistance between baculovirus and chemical insecticides or *Bt* toxins in *H. armigera*, *Spodoptera frugiperda* (J. E. Smith), *Helicoverpa punctigera* (Wallengren), *Chloridea virescens* (F.), *Chrysodeixis includens* (Walker) (Lepidoptera: Noctuidae), and *Plutella xylostella* (L.) (Lepidoptera: Plutellidae) (Ignoffo and Roush 1986; Raymond et al. 2006; Bentivenha et al. 2018; Godoy et al. 2019; Windus et al. 2021). These findings indicate that baculoviruses can be integrated with other control tactics to delay the evolution of resistance to chemical insecticides and *Bt* toxins. Nonetheless, a few studies have demonstrated that resistance to baculoviruses can also evolve, as observed in *S. frugiperda* to SfMNPV and *Cydia pomonella* (L.) (Lepidoptera: Tortricidae) to CpGV (Fuxa et al. 1988; Asser-Kaiser et al. 2011).

Characterizing the susceptibility of geographically distinct populations of a target pest to chemical or biological insecticides provides essential reference data for proactive resistance-monitoring programs (Tabashnik et al. 2014). These data allow the establishment of diagnostic concentrations to detect early shifts in susceptibility or changes in resistance allele frequencies (Sims et al. 1996). Consequently, before the introduction of any new insecticide—including biological products such as AcMNPV—it is important to establish baseline susceptibility of target species to support future resistance monitoring and resistance management strategies.

Given the promising adoption of AcMNPV-based bioinsecticide against *R. nu* in Brazil, the objectives of this study were to (a) characterize the susceptibility of Brazilian populations of *R. nu* to AcMNPV, (b) establish a diagnostic concentration for resistance-monitoring programs, and (c) investigate cross-resistance between AcMNPV and Cry1Ac *Bt* toxin in *R. nu*.

## Material and methods

### Insects

**Fig. 1:**
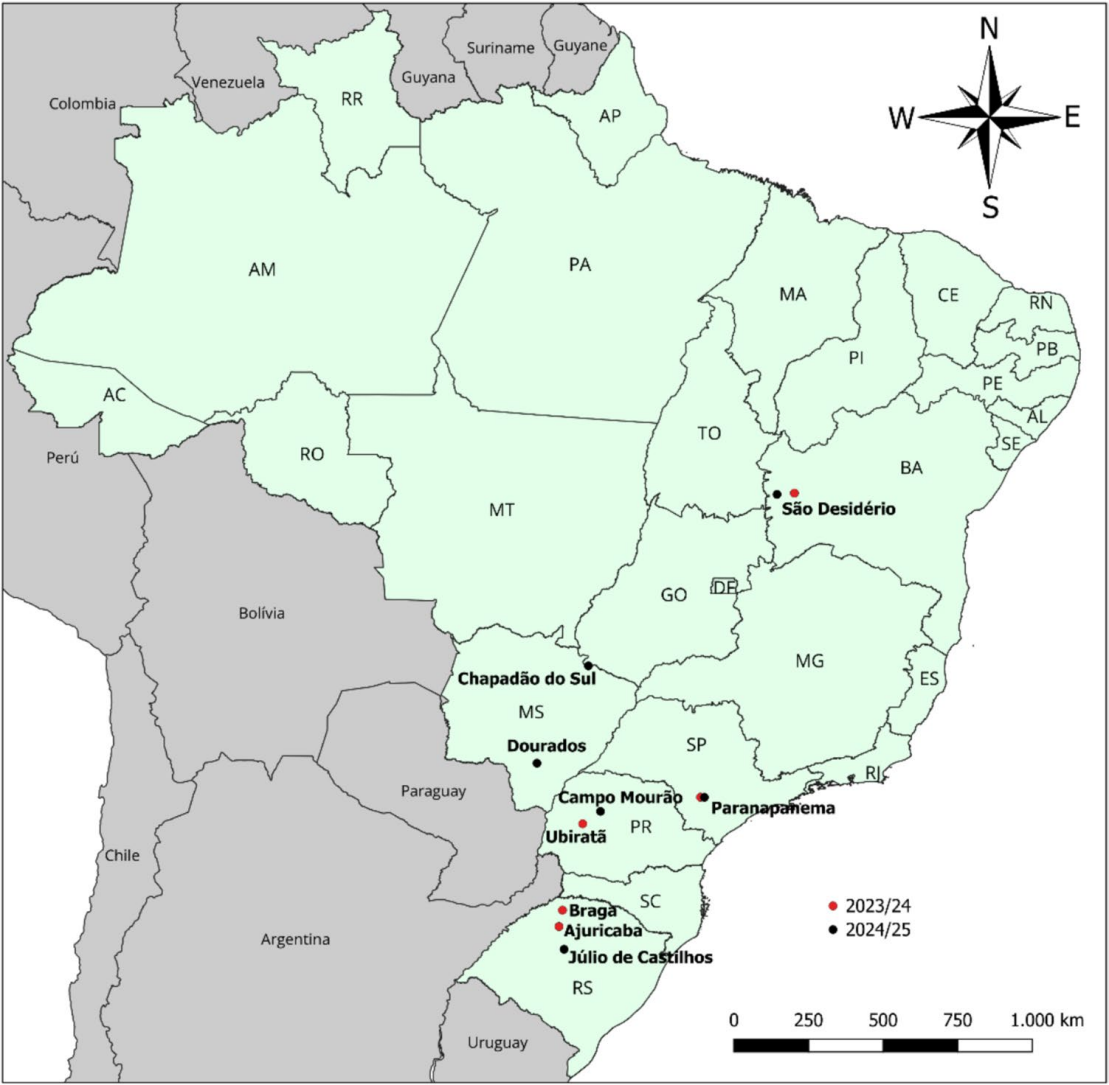
Sampling sites of *Rachiplusia nu* from soybean in Brazil

To characterize the susceptibility of *R. nu* to AcMNPV, 11 populations were collected in main soybean-producing regions where *R. nu* has been reported in Brazil (Godói et al. 2025). Collections were conducted in five Brazilian states during the 2023/24 and 2024/25 soybean seasons, with 300–1200 larvae sampled/collection site (Table 1; Fig. 1). After collection, larvae were transported to the laboratory and individually placed in 50-mL plastic cups containing 3 mL of an artificial diet based on white beans, wheat germ, and yeast (Greene et al. 1976), where they remained until completing the larval stage. In addition to these field populations, we also included in the assessments a susceptible population of *R. nu*. This population was collected in non-*Bt* soybean in Santana do Livramento, RS, Brazil (30°50′15″S; 55°23′43″W), in February 2022 and has been maintained for > 16 generations under laboratory conditions without exposure to insecticides or *Bt* toxins. This population was previously used as a source of susceptible insects in studies with chemical insecticides and *Bt* traits (Godoy et al. 2024; Reis et al. 2024).

**Table 1:** Identification of *Rachiplusia nu* populations collected in Brazilian soybean fields and used to assess the susceptibility to AcMNPV

Collection site	Latitude (S)	Longitude (W)	Date of collection
2023/24 soybean season			
Braga, RS	27°37′52″	53°41′59″	February 2024
Ajuricaba, RS	28°13′46″	53°49′42″	February 2024
Ubiratã, PR	24°28′23″	52°57′45″	January 2024
Paranapanema, SP	23°30′10″	48°39′33″	December 2023
São Desidério, BA	12°23′50″	45°13′54″	March 2024
2024/25 soybean season			
Júlio de Castilhos, RS	29°03′45″	53°38′33″	November 2024
Campo Mourão, PR	24°01′15″	52°18′44″	February 2025
Paranapanema, SP	23°30′24″	48°31′06″	January 2025
Chapadão do Sul, MS	18°41′56″	52°45′19″	January 2025
Dourados, MS	22°15′29″	54°38′01″	January 2025
São Desidério, BA	12°26′40″	45°51′58″	January 2025

To evaluate patterns of cross-resistance between AcMNPV and Cry1Ac *Bt* toxin, two *R. nu* populations were also tested: (i) a population with resistance to Cry1Ac and (ii) the susceptible population described above. The Cry1Ac-resistant population was derived from a field-collected population, as described in detail by Reis et al. (2024). This population exhibited > 736-fold resistance to Cry1Ac in diet-overlay bioassays used to characterize the inheritance of resistance. Following the initial selection, the Cry1Ac-resistant strain has been maintained in the laboratory for approximately 16 generations, with selection pressure applied using Cry1Ac soybean leaves every two generations. When the studies reported here were initiated, this population showed > 92% survival on Cry1Ac soybean.

### Baseline susceptibility of* R. nu *to AcMNPV in diet-overlay bioassays

To characterize the susceptibility of *R. nu* to AcMNPV, diet-overlay bioassays were conducted with six field populations—Braga and Ajuricaba (RS), Ubiratã (PR), Paranapanema (SP), Dourados (MS), and São Desidério (BA)—collected from 2023 to 2025 (Table 1; Fig. 1) along with the susceptible population described above. Bioassays were performed in 24-well acrylic plates (Costar®, São Paulo, SP, Brazil) containing 1 mL/well of an artificial diet (Greene et al. 1976), which was prepared without formaldehyde and antibiotics. After plate preparation, a commercial biopesticide containing AcMNPV (Disseminate®, 7.50 × 10^9^ occlusion bodies (OBs)/mL; AgBiTech, Fort Worth, TX, USA) was diluted in distilled water to prepare the range of concentrations to be tested. A surfactant, Triton X-100 (Sigma–Aldrich, São Paulo, SP, Brazil), was added to each concentration at 0.1% (v/v) to obtain a uniform spread when the solution was applied to the diet surface. Then, 6–8 concentrations of AcMNPV (1 × 10^7^ to 5.6 × 10^8^ OBs/mL) were applied to the diet surface (30 µL/well over a surface area of 1.88 cm^2^), using an automatic replication pipette (Multipette® E3x; Eppendorf, São Paulo, SP, Brazil). The control treatment was composed of distilled water + surfactant. After a drying period ($\sim$30 min) inside a laminar air flow cabinet, a single early-L2 larva ($\sim$0.5 long and 4 days after hatching) of *R. nu*, from F_1_ to F_3_ generations, was transferred to each well. After larval infestation, plates were closed with a plate cover and stored in a climatic chamber at 25 ±  2°C, 65% ± 10% relative humidity, and a 14:10-h light/dark photoperiod. The experimental design was completely randomized with two replicates of 24 larvae/replicate, totaling 48 larvae tested/concentration/population or control treatment. Mortality was assessed 7 days post-infection: larvae that did not move after being touched with a tweezer were considered dead.

### Establishing a diagnostic concentration of AcMNPV for resistance monitoring

The bioassay method for defining the diagnostic concentration of AcMNPV for resistance monitoring was as described above. For this study, the field populations of *R. nu* collected during the 2024/25 soybean season were used (Table 1). In the bioassays, 240 early-L2 larvae from F_1_ to F_3_ generations of each population were tested (10 replicates of 24 larvae/population). Mortality assessments were the same as described in the previous section.

### Assessment of cross-resistance between AcMNPV and Cry1Ac Bt toxin in* R. nu*

To perform this study, early-L2 larvae of Cry1Ac-resistant and -susceptible populations (described in detail in the “Insects” section) were exposed to infection by AcMNPV in diet-overlay bioassays. The bioassay procedures, environmental conditions, experimental design, and mortality assessment followed those described for the baseline susceptibility study.

### Statistical analysis

The concentration–mortality data from bioassays with field populations of *R. nu* and from the cross-resistance study were analyzed using a Generalized Linear Model (GLM) with a binomial distribution and a probit link function using R software (R Core Team 2021). Goodness-of-fit tests (χ^2^ tests) were performed using the *hnp* package (Moral et al. 2017) to verify whether the data fit the assumptions of the probit model. If the χ^2^ values predicted by the model did not differ significantly (*p* > 0.05) from those of the observed data, the model was assumed to fit the data. Then, LC_50_ and LC_90_ (concentrations lethal to 50% and 90% of the insects, respectively) and respective 95% confidence intervals (CIs) were estimated with the *dose.p* function from the MASS package in R software (R Core Team 2021).

To estimate a diagnostic concentration of AcMNPV for resistance monitoring in *R. nu*, the entire concentration–mortality dataset of the six field populations used in baseline susceptibility bioassays was pooled and analyzed jointly, as suggested by Muraro et al. (2019). In this analysis, the LC_99_ and 95% CI were estimated using a GLM with a binomial distribution and *cloglog* link function in R software (R Core Team 2021). Through this approach, the diagnostic concentration of AcMNPV was identified based on the estimated LC_99_ value. To validate the diagnostic concentration of AcMNPV for resistance monitoring, the mortality percentages of populations of *R. nu* were compared by the Tukey adjustment test (*p* > 0.05) using the *emmeans* package in R software (R Core Team 2021).

## Results

### Baseline susceptibility of* R. nu *to AcMNPV

The range of AcMNPV concentrations tested against early-L2 larvae from geographically distinct populations of *R. nu* from Brazil resulted in mortality ranging from 8.3% to 97.9%. For six field populations of *R. nu*, the LC_50_ of AcMNPV ranged from 1.9 × 10^7^ (São Desidério, BA) to 7.9 × 10^7^ (Ubiratã, PR) OBs/mL (Table 2). The LC_90_ of AcMNPV ranged from 2.8 × 10^8^ (Ajuricaba, RS) to 1.3 × 10^9^ (Braga, RS) OBs/mL. The variations in susceptibility demonstrated the existence of < 4.2- and < 4.7-fold differences in susceptibility to AcMNPV, based on LC_50_ and LC_90_ values, respectively. Our results also indicate that most field populations had comparable susceptibility or were more susceptible to AcMNPV than the susceptible population used as the standard for susceptibility comparisons (Table 2).

**Table 2:** Lethal-concentration (LC; occlusion bodies (OBs)/mL) responses of Brazilian populations of *Rachiplusia nu* to AcMNPV in diet-overlay bioassays

Population (2023/24 season)	*n*	Fit of probit lines			LC_50_ (95% CI)^d^	LC_90_ (95% CI)^d^
		Slope ± SE^a^	*χ*^2^ (*df*)^b^	*p*^c^		
Susceptible	384	1.65 ± 0.17	3.22 (5)	0.67	6.8 × 10^7^ (5.5 × 10^7^–8.3 × 10^7^)	4.0 × 10^8^ (2.7 × 10^8^–6.1 × 10^8^)
Braga, RS	384	1.00 ± 0.14	3.17 (5)	0.67	6.9 × 10^7^ (4.9 × 10^7^–9.7 × 10^7^)	1.3 × 10^9^ (6.0 × 10^8^–2.9 × 10^9^)
Ajuricaba, RS	384	1.43 ± 0.16	8.31 (5)	0.14	3.6 × 10^7^ (2.7 × 10^7^–4.8 × 10^7^)	2.8 × 10^8^ (1.9 × 10^8^–4.2 × 10^8^)
Ubiratã, PR	384	1.29 ± 0.15	2.77 (5)	0.74	7.9 × 10^7^ (6.1 × 10^7^–1.0 × 10^8^)	7.8 × 10^8^ (4.5 × 10^8^–1.3 × 10^9^)
Paranapanema, SP	336	1.63 ± 0.19	1.84 (4)	0.77	6.8 × 10^7^ (5.3 × 10^7^–8.7 × 10^7^)	4.2 × 10^8^ (2.8 × 10^8^–6.2 × 10^8^)
Dourados, MS	384	1.70 ± 0.17	5.58 (5)	0.35	7.0 × 10^7^ (5.6 × 10^7^–8.7 × 10^7^)	4.0 × 10^8^ (2.8 × 10^8^–5.7 × 10^8^)
São Desidério, BA	336	1.06 ± 0.15	2.47 (4)	0.65	1.9 × 10^7^ (1.2 × 10^7^–3.1 × 10^7^)	3.1 × 10^8^ (1.7 × 10^8^–5.5 × 10^8^)

^a^*SE* standard error

^b^*df* degrees of freedom

^c^*p* > 0.05 in the goodness-of-fit test indicated good fit to the probit model

^d^LC_50_ and LC_90_ are the concentrations of AcMNPV required to kill 50% and 90% of larvae, respectively, after 7 days of exposure

For estimating the diagnostic concentration of AcMNPV, the concentration–mortality datasets of the field populations were pooled and analyzed jointly. By the joint analysis, the LC_99_ (concentration required to kill 99% of larvae) was estimated to be 1.6 × 10^9^ (95% CI 1.2 × 10^9^ to 2.1 × 10^9^) OBs/mL (*n* = 2208; slope ± SE = 1.28 ± 0.07; χ^2^ = 4.29; *df* = 6; *p* = 0.64). The LC_99_ value was established as the potential diagnostic concentration to monitor *R. nu* resistance to AcMNPV in Brazil.

### Validation of a diagnostic concentration of AcMNPV for resistance monitoring

The mortality rates of field populations of *R. nu* exposed to the diagnostic concentration based on the LC_99_ of AcMNPV (1.6 × 10^9^ OBs/mL) ranged from 97.9% to 99.6%, not differing significantly from the mortality of the susceptible population (100% mortality) (*F* = 1.10; *df* = 6, 149; *p* = 0.36) (Table 3).

**Table 3:** Mortality of Brazilian populations of *Rachiplusia nu* exposed to the diagnostic concentration of AcMNPV in diet-overlay bioassays

Population (2024/25 season)	Number tested	Number of dead	% mortality (± SE)^a,b^
Susceptible	240	240	100.0 ± 0.0 a
Júlio de Castilhos, RS	240	239	99.6 ± 0.4 a
Campo Mourão, PR	240	236	98.3 ± 0.8 a
Paranapanema, SP	240	235	97.9 ± 0.8 a
Dourados, MS	240	238	99.2 ± 0.6 a
Chapadão do Sul, MS	240	238	99.2 ± 0.6 a
São Desidério, BA	240	238	99.2 ± 0.6 a

^a^*SE* standard error

^b^No significant differences in mortality between susceptible and field populations of *R. nu* were detected as indicated by Tukey’s test (*p* > 0.05)

### Cross-resistance between AcMNPV and Cry1Ac

The LC_50_ and LC_90_ of AcMNPV against early-L2 larvae of *R. nu* from Cry1Ac-resistant and -susceptible populations were similar (Table 4), suggesting that there is no cross-resistance between AcMNPV and Cry1Ac *Bt* toxin in *R. nu*.

**Table 4:** Lethal-concentration (LC; occlusion bodies (OBs)/mL) response of the susceptible and Cry1Ac-resistant populations of *Rachiplusia nu* to AcMNPV in diet-overlay bioassays

Population	*n*	Fit of probit lines			LC_50_ (95% CI)^d^	LC_90_ (95% CI)^d^	RR^e^
		Slope ± SE^a^	*χ*^2^ (*df*)^b^	*p*^c^			
Susceptible	336	1.63 ± 0.18	1.41 (4)	0.84	6.2 × 10^7^ (4.9 × 10^7^–7.8 × 10^7^)	3.8 × 10^8^ (2.5 × 10^8^–5.8 × 10^8^)	-
Cry1Ac-resistant	384	1.35 ± 0.17	3.41 (5)	0.64	4.5 × 10^7^ (3.4 × 10^7^–6.0 × 10^7^)	4.0 × 10^8^ (2.5 × 10^8^–6.4 × 10^8^)	0.7

^a^*SE* standard error

^b^*df* degrees of freedom

^c^*p* > 0.05 in the goodness-of-fit test indicated good fit to the probit model

^d^LC_50_ and LC_90_ are the concentrations of AcMNPV required to kill 50% and 90% of larvae tested, respectively, after 7 days of exposure

^e^Resistance ratio (RR) = (LC_50_ of Cry1Ac-resistant strain)/(LC_50_ of susceptible strain)

## Discussion

In this study, we documented that early-L2 larvae of populations of *R. nu* were susceptible to AcMNPV applied to the surface of an artificial diet. The variation in susceptibility to AcMNPV, in terms of LC_50_ and LC_90_, was < 4.7-fold. We believe that the variation in susceptibility in *R. nu* populations from Brazil to the AcMNPV-containing bioinsecticide does not reflect a previous exposure to selection pressure but instead probably represents natural interpopulation variability in susceptibility. Similar levels of variation in susceptibility to baculoviruses were also reported in Brazilian populations of *C. includens*—another Plusiinae pest of soybean—to ChinNPV (5.5-fold), in *S. frugiperda* to SfMNPV (2.1-fold), and in *H. armigera* to HearNPV (7.3-fold) (Bentivenha et al. 2018; Muraro et al. 2019, 2022).

The high susceptibility of Brazilian populations of *R. nu* to AcMNPV appears to be associated with species-specific traits and historical management practices. This pattern likely reflects an inherent susceptibility resulting from the recent introduction of this biopesticide in Brazil in 2023 and the low exposure to selection pressure by baculovirus-containing insecticides. The high susceptibility to AcMNPV may also be influenced by the recent population expansion of *R. nu* in soybean in Brazil (Horikoshi et al. 2021a; Godói et al. 2025). Historically, *R. nu* was considered a minor soybean pest, occurring at low levels in the mid-south region of the country, which likely resulted in low exposure to insecticides applications (Godoy et al. 2024). Consistent with this interpretation, Brazilian populations of *R. nu* have also been reported to be highly susceptible to several chemical insecticides with distinct modes of action (Godoy et al. 2024). Therefore, the use of AcMNPV-based biopesticides, according to label recommendations, represents a valuable tool for controlling *R. nu* in soybean production systems in Brazil.

The AcMNPV-based insecticide was also lethal against early-L2 larvae of *R. nu* with resistance to Cry1Ac *Bt* toxin, indicating no cross-resistance between these different bioinsecticides. Similarly, no cross-resistance between baculoviruses and *Bt* proteins was reported in *S. frugiperda*, *P. xylostella*, or *H. punctigera* (Raymond et al. 2006; Bentivenha et al. 2018; Windus et al. 2021) or between baculoviruses and chemical insecticides in *C. includens*, *S. frugiperda*, *C. virescens*, and *H. armigera* (Ignoffo and Roush 1986; Bentivenha et al. 2018; Godoy et al. 2019; Muraro et al. 2022). The lack of cross-resistance between AcMNPV and Cry1Ac in *R. nu* indicates that this baculovirus product represents a valuable tool for use against this soybean pest in rotation with chemical insecticides and *Bt*-based bioinsecticides. According to Tabashnik (1989), the strategy of rotating insecticides with distinct modes of action is only effective if there is no cross-resistance between the control agents used in rotation.

The lack of cross-resistance between AcMNPV and Cry1Ac *Bt* toxin was expected owing to their distinct modes of action, even though both act in the insect midgut. The baculoviruses produce OBs that are ingested by larvae and then dissolved in the alkaline midgut fluids, releasing virions that establish the initial infection. Once the virus enters the nucleus of midgut cells and replicates, a secondary infection begins when new viruses are produced and infect other tissues and organs, leading to the death of the larvae (Hoover et al. 2002; Song et al. 2016; Boogaard et al. 2017). In contrast, Cry toxins cause the lysis of midgut epithelial cells by inserting into the cell membranes and forming pores; this results in release of the cell contents, which provide a germination medium for spores, thus leading to severe septicemia and insect death (Bravo et al. 2007).

In summary, our findings demonstrate that the AcMNPV-based bioinsecticide is a feasible tool for controlling *R. nu* in soybean in Brazil. However, this baculovirus should be used in rotation with insecticides having other modes of action because insects are also able to evolve resistance to baculoviruses (Fuxa et al. 1988; Asser-Kaiser et al. 2011). Therefore, we recommend regular monitoring of the susceptibility of *R. nu* to AcMNPV with the diagnostic concentration determined in the current study. This will allow early detection of shifts in susceptibility to AcMNPV, providing opportunities for resistance management.

## Conclusions

A low interpopulation variation in the susceptibility of *R. nu* to AcMNPV was detected in Brazil. The diagnostic concentration of AcMNPV determined in the current study caused high mortality of field populations of *R. nu* and is suggested for use in resistance-monitoring programs. No evidence of cross-resistance was detected between AcMNPV and Cry1Ac *Bt* toxin, suggesting that this baculovirus-based insecticide can be a valuable tool for managing *R. nu* in soybean.

## Data Availability

The data used in the present work are available from the authors upon reasonable request.
